# Raising awareness of cognitive biases during diagnostic reasoning

**DOI:** 10.1007/s40037-016-0274-4

**Published:** 2016-05-23

**Authors:** Kaylee van Geene, Esther de Groot, Carmen Erkelens, Dorien Zwart

**Affiliations:** Newzoo BV, Amsterdam, The Netherlands; Julius Centre for Health Sciences and Primary Care, University Medical Center Utrecht, Utrecht, The Netherlands

**Keywords:** Cognitive bias, General practitioners, Salient distracting features, Experiential learning

## Abstract

**Introduction:**

Bias in diagnostic reasoning can potentially lead to severe consequences. We explored how to design an experiential learning workshop in a general practice clerkship to raise awareness on bias.

**Method:**

A group of 12 students was split into two groups. Both groups ‘diagnosed’ two patients in two case studies. Only one group, without them knowing, were given a case including salient distracting features. The whole group discussed the influence of these distractors. In the second round all students had salient distracting features in their case descriptions but only one group had a debiasing tool, a checklist to reconsider their first diagnosis, which they discussed in the final large group discussion.

**Results:**

Students were misled by salient distracting features and thus experienced how one small difference in a case description may lead to a different diagnosis, due to bias. The debiasing tool was regarded with scepticism. Afterwards, students indicated that, thanks to experiencing bias themselves, they felt better equipped to recognize the risk of bias.

**Conclusions:**

An experiential learning approach with case studies containing salient distracting features seems to be a viable method to learn about bias in a general practice clerkship.

## Introduction

Diagnostic decision-making is difficult, with a variety of pitfalls, one of which is diagnostic error through cognitive bias [1, 2]. Cognitive biases in diagnostic decision-making are inclinations to use mental shortcuts to develop a diagnosis, which may lead to an inaccurate interpretation of the patient’s complaint with potentially severe consequences [3, 4]. Confirmation bias, anchoring bias and premature closure are just some examples of many biases that have been described [5, 6]. Salient distracting features are findings in a case description that tend to grab the attention because they are typical for a particular disease, but are unrelated to the actual problem. Salient distracting features can misdirect diagnostic reasoning and lead to mistakes [1]. Researchers advocate the need for more attention in clinical practices to ways to reduce the influence of cognitive biases during diagnostic decision-making (debiasing) [5]. The process of ‘debiasing’ general practitioners (GPs), who have to make a diagnosis from a wide range of possibilities where mistakes could easily be made, should start early in their training [7].

The University Medical Center Utrecht (UMCU) wondered how to design such a training. How can a workshop within the general practice clerkship be designed to help students recognize and reduce the influence of bias in diagnostic reasoning? *Telling* students about the existence of biases and teaching facts is not effective in learning to recognize situations in which bias may influence decisions [8]. What is needed is to *experience* the encounter of errors as a result of bias, in a setting where students can make mistakes without serious consequences [9]. From studies it is known that in order to realize the importance of bias, GPs have to experience the consequences for themselves [7]. Therefore, the design of our workshop was about experience of bias, being motivated by this, and about working with a debiasing tool, a checklist that presses for ‘second thoughts’. The most important goal was to raise awareness of bias. We designed an experiential learning workshop where students experience, reflect on and discuss bias.

## Methods

**Fig. 1:**
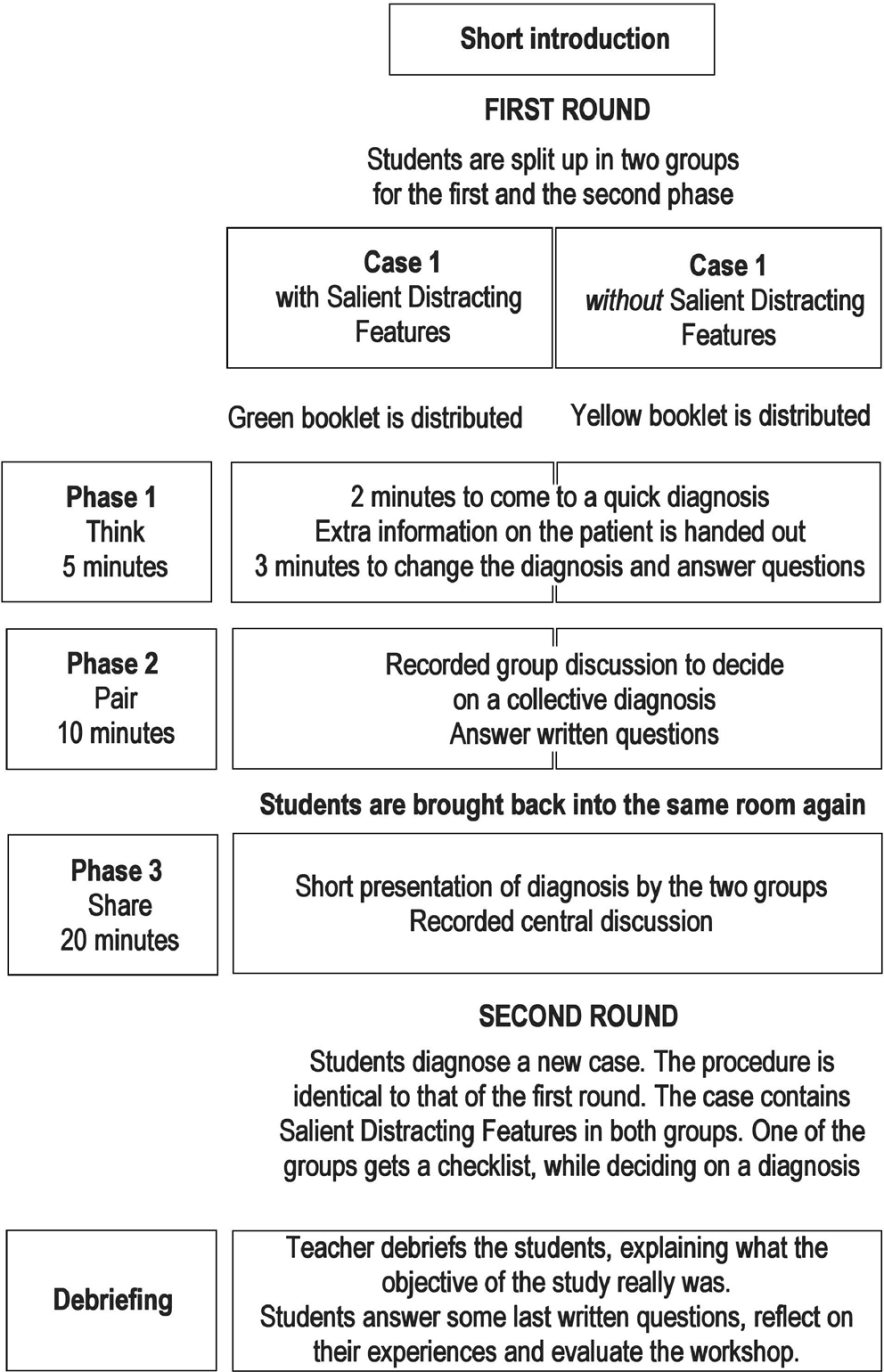
Lay-out of the workshop

All participants in the three subsequent runs of our workshop were students in their fifth year of medical training, taking part in the UMCU general practice clerkship; each week a different group took part. In their schedule a workshop about emergency medicine in general practice was announced. At the start, students were given a booklet which contained a case based on real-life emergency telephone calls either with salient distracting features, such as stomach pain which is unrelated to cardiovascular disease, or without such distracting features. In the booklet participants had to answer open questions during and after the experiment, for example about how certain they were about their diagnosis, what they thought they had learned and what they felt about this workshop. The whole workshop comprised three phases (Fig. 1): individual phase 1, small group phase 2 and large group phase 3. Together with two GPs we developed a teaching guide. The cases, the questions, the guide and the checklist are available from the authors upon request. This workshop, which was held in June and July 2015, is part of a PhD project. The METC approved the PhD project.

## Results

The participants enjoyed the workshop, labelling this experience as instructive and insightful. Discussing patient cases in groups in this way gave them a different outlook on diagnostic reasoning. Experiencing that bias can evoke serious adverse events made them more aware of the pitfalls in their thinking and they were less confident in their diagnosis on the second case. The results indicate that two versions of a case – with or without distractors – were successful in misleading the participants. Even though they were more aware of bias in the second round, they were again misled by salient distracting features.

## First run of the workshop

In run 1, case 1, students were inclined to discuss the medical aspects of the case instead of the influence of bias. Any doubts on the differential diagnosis raised by co-participants were brushed aside, making them conform to the opinion of the group. Bias got little attention because the teacher did not mention this and thus participants missed the fact that this was the real goal of the workshop. Participants did not seem convinced that they had been misled by the salient distracting features (in phase 3, they stated: ‘*The reflective way of diagnosing is already inherent in our thinking. We already do this, this is how we are being trained*.’).

Despite the consequences of their actions, which theoretically would have led to the death of two patients, we noticed little concern in our participants, indicated by some of the remarks made: ‘*I did not think something really bad was going on*’, ‘*I’m not too worried about this patient*’. Our own conclusion after the first run was that experiencing bias alone is not sufficient and it is important to help students to talk explicitly about it. To motivate them the teacher should tell them early on about the real life course of the patient on whom the case was built.

## Second and third run

In the second and third run, the approach of the workshop was slightly altered, based on the experiences in the first run, and taught by a different teacher. In phase 3 the teacher focused on the salient distracting features present in the case; how these misleading pieces of information can influence thinking and that this phenomenon is called cognitive bias. This time participants were interested in the differences between the groups’ case descriptions. During phase 3 they stated that these small and subtle changes, which are expected to be present in many cases, can make a big difference to your thinking process and to your diagnosis, as shown by the following remarks: ‘*It is entirely up to how the patient presents him or herself and exactly what you ask*.’ and ‘*Sometimes this makes it easier to lead you down a different path*.’ Participants seemed quite shaken at hearing this patient had actually passed away, because the ‘real life’ doctors had made the same mistakes as they did.

In case 2 the participants were more cautious and watchful in both runs, taking a defensive approach because of the consequences in the first case (reflected in the following statements: ‘*Because we were misled on the first case, I am a bit more defensive in this second case*’, ‘*I am afraid of letting someone die again*’). The group that had not experienced the effect of the salient distracting features in case 1 (because these features were absent) felt in case 2 that the distractors pushed them into a certain direction. Apparently, the group discussion after case 1 (where they had only observed the phenomenon of bias due to salient distracting features and the effect it had on the other group) sensitized them to the risk of bias.

In both runs participants showed more awareness and asked for more experiential learning about bias, with more diverse cases including varying amounts of bias. They were able to recognize different forms of bias, were able to discuss these and reflect on the importance of being aware of them.

## Discussion

Participants showed they were capable of recognizing risk factors on bias after a first case, but this did not prevent diagnostic mistakes in the second case. In this educational setting it was more important to learn to be more aware of bias and its consequences rather than reach the right diagnosis. Instructions for the teachers in this respect had been included in a teachers’ guide which needs to be discussed with the teacher in advance because ‘accepting the wrong diagnosis’ may feel counter-intuitive for them. From the students’ remarks in run 2 and 3, we deduced that it is important to make participants aware of the realism of the cases and the omnipresence of risk factors for bias. In the central discussion, the teacher has to mention explicitly the inclusion of salient distracting features and discuss how these distractors can lead to bias. The workshop helps students to be more aware of how they look at the information in the case description. Participants were able to recognize, discuss and reflect on a variety of biases that were evoked in the patient cases. The differences between the first and following times the workshop ran could be partially explained by better preparation (discuss the teachers’ guide in advance) and by more teaching experience on the part of the teacher who supervised the second and third run. The workshop requires a delicate balance of letting participants experience the effect and making the effect explicit for them.

## Conclusion

This experiential learning workshop showed an increase in the awareness of medical students on bias and motivation to pay attention to bias. The didactic approach requires careful preparation to ensure that participants are not expecting to talk about bias and to ensure that teachers know that making mistakes is part of the design. It is not possible to remove all biases from diagnostic reasoning processes, but students should be more aware of the existence and the influence bias can have. During the workshop students experience that they do actually fall for the distractions of the salient distracting features, which is probably more effective than only *telling* them about these pitfalls, but we need to and will perform additional studies to understand the learning process better.
